# Shining light on spindle positioning

**DOI:** 10.7554/eLife.38748

**Published:** 2018-07-09

**Authors:** Andrea Serra-Marques, Sophie Dumont

**Affiliations:** 1 Department of Cell and Tissue BiologyUniversity of CaliforniaSan FranciscoUnited States; 2 Department of Cellular and Molecular PharmacologyUniversity of CaliforniaSan FranciscoUnited States

**Keywords:** spindle positioning, cortical pulling forces, dynein, NuMA, optogenetic control, Human

## Abstract

Optogenetic approaches are leading to a better understanding of the forces that determine the plane of cell division.

**Related research article** Okumura M, Natsume T, Kanemaki MT, Kiyomitsu T. 2018. Dynein-Dynactin-NuMA clusters generate cortical spindle-pulling forces as a multi-arm ensemble. *eLife* **7**:e36559. doi: 10.7554/eLife.36559

Cell division is essential to grow, maintain and repair an organism. To evenly distribute a copy of its genetic material between the new cells, the cell uses a machine called the spindle. This machine is made up of protein filaments known as microtubules: some of them attach to the chromosomes, while others known as astral microtubules anchor the spindle to the cell periphery at the cell cortex. The position of the spindle within the cell directs where the cell divides and, therefore, determines the size – and in polarized cells the fate – of the two new cells (Lu and Johnston, 2013).

To position the spindle within the cell, molecular motors at the cell cortex pull on the astral microtubules (Grill and Hyman, 2005). In human cells undergoing symmetric cell division, signals travel from the chromosomes and the spindle to the cell cortex in order to dynamically adjust the force being generated according to the position of the spindle (Kiyomitsu, 2015).

The force-generating machinery at the cortex consists of an evolutionary conserved set of proteins, including a long protein called NuMA, a motor protein called dynein, and a multiprotein complex called dynactin that is needed to activate dynein (Galli and van den Heuvel, 2008; Kotak et al., 2012; Gönczy, 2008). NuMA recruits dynein and dynactin to the cell cortex, and dynein generates the force that is needed to pull the spindle towards the cortex. However, there is much that we do not understand about spindle positioning. For example, is dynein alone sufficient for force generation? And how is it possible to produce persistent forces when the machinery responsible is mobile and the astral microtubules are dynamic? Now, in eLife, Tomomi Kiyomitsu from Nagoya University and colleagues – including Masako Okumura as the first author – report new insights into how the force-generating machinery is regulated and organized to pull on the spindle (Okumura et al., 2018).

Okumura et al. used a light-controllable system (Guntas et al., 2015) to manipulate where and when the various proteins in the machinery were recruited to the cell cortex. This enabled them to establish a direct cause-and-effect relationship between the location of proteins at a specific time and a subsequent spindle movement in that direction. The researchers recruited NuMA directly to the cell membrane, bypassing the canonical recruitment pathway (which involves proteins called Gαi and LGN; Figure 1A, top). This revealed that the complex formed by NuMA, dynein and dynactin is sufficient to initiate the pulling force from the cortex, independent of the proteins that normally recruit them to the cortex. They also showed that dynein cannot generate enough force to pull the spindle on its own – NuMA must also be recruited (Figure 1A, bottom). This synergizes with findings from a recent study using worm embryos (Fielmich et al., 2018): targeting the worm equivalent of NuMA with light suffices to move the spindle, while targeting dynein alone does not. This indicates that NuMA may have other roles beyond simply recruiting dynein to the cortex. What, then, does it do?

**Figure 1.**
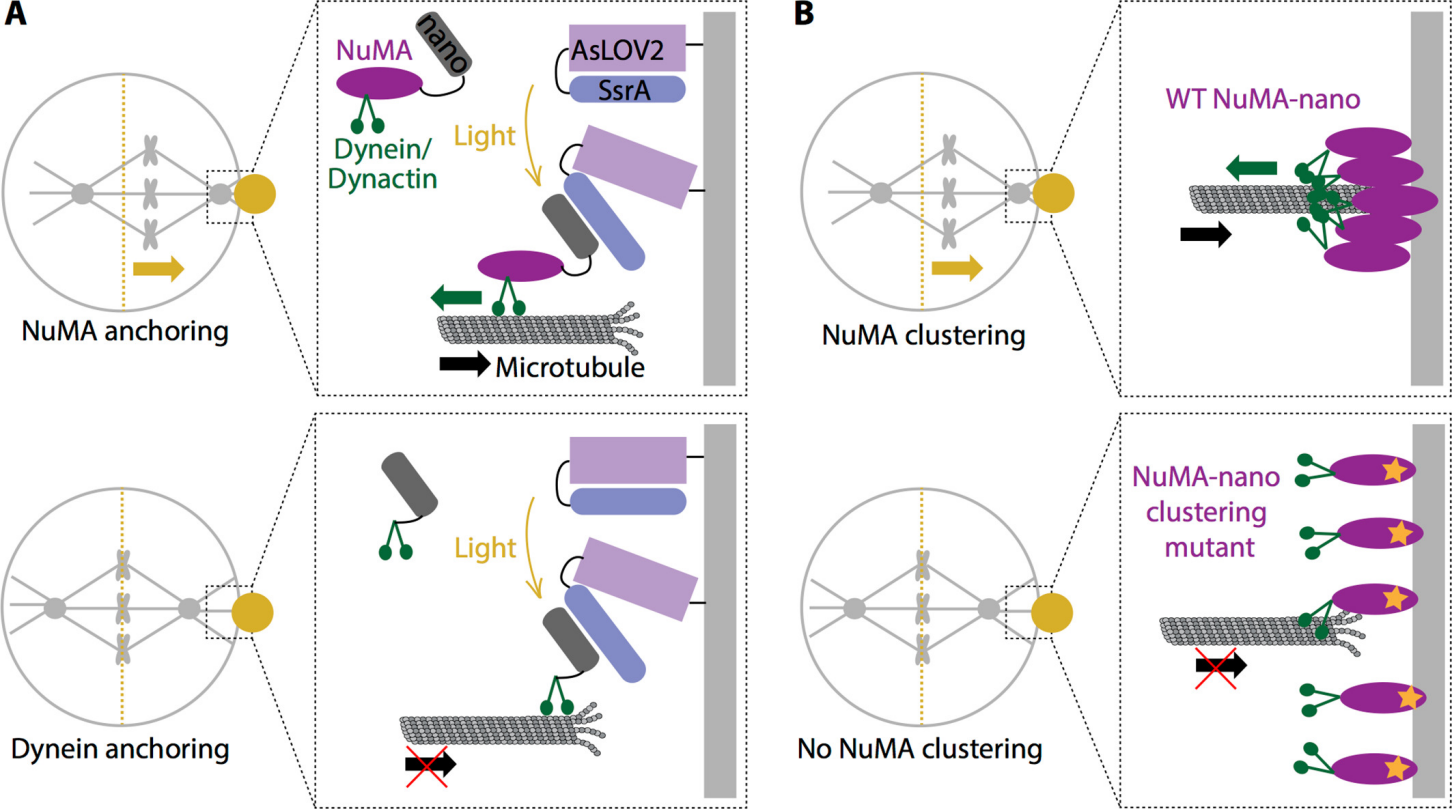
Force generation at the cell cortex and spindle positioning. (**A**) Okumura et al. built a system in which a light-sensitive protein module (AsLOV2; lilac: SsrA; blue) was expressed at the cortex (light grey), allowing the induced recruitment of nano (dark grey) fused to either NuMA (purple; top) or dynein (green; bottom). When a specific region in the cell cortex (gold circle) is illuminated, NuMA-nano binds to the light-sensitive module and is recruited to the cortex, which leads to the spindle moving (gold arrow) towards the illuminated region (top). When the same experiment is repeated by directly targeting dynein to the cortex with light, the spindle does not move (bottom). Thus, anchoring NuMA to the cortex is sufficient for spindle pulling, but anchoring dynein is not. (**B**) Okumura et al. also examined how wild type (WT) NuMA (left) and a mutant NuMA (right) are organized at the cortex. When the system was illuminated, WT NuMA formed clusters at the cortex, which led to the spindle moving (gold arrow) towards the illuminated region (top). However, when mutant NuMA (purple with orange star) that does not self-assemble is used, neither clustering nor spindle movement (bottom) occur, indicating that NuMA’s ability to cluster and ‘concentrate’ force at the cortex is required for spindle pulling. For simplicity the light-sensitive module is not shown in (**B**).

The experiments revealed that a specific region on NuMA, similar to a motif found in proteins that activate dynein, is required in order to recruit dynein. Together with NuMA being necessary for force generation, this finding suggests that NuMA could activate dynein, as if it turned the key in dynein’s engine to make it persistently 'walk'. However, further work is needed to confirm this. The results also showed that both the long coiled-coil structure of NuMA and the region, or domain, that binds to the microtubules are essential for the cortex to pull on the spindle. NuMA’s ability to bind microtubules could help dynein hold onto the microtubules, while its long structure may help the cortical machinery to capture the microtubules and grasp on to them in this dynamic environment.

Finally, Okumura et al. identified a region on NuMA that promotes the assembly of clusters with dynein and dynactin at the cell cortex, which in turn is necessary to promote the pulling forces on the spindle (Figure 1B). Clustering many dynein motors could help them to capture microtubules and to tug on them more efficiently. Okumura et al. offer a provocative model in which NuMA could assemble a ring-like structure at the cortex, creating a platform similar to the microtubule-binding machine located at the chromosomes. Going forward, the precise architecture and function of these force-generating clusters at the cell cortex will be important questions to address.

In summary, NuMA appears to be multi-talented, using its different domains and functions to promote robust force generation and spindle positioning. It has long ‘fingers’, generates passive force by binding microtubules, and recruits, clusters, and may even turn on motor proteins, generating active force. These may be generalizable principles for how cells build dynamic interfaces that must robustly generate force.
